# SBML Level 3: an extensible format for the exchange and reuse of biological models

**DOI:** 10.15252/msb.20199110

**Published:** 2020-08-26

**Authors:** Sarah M Keating, Dagmar Waltemath, Matthias König, Fengkai Zhang, Andreas Dräger, Claudine Chaouiya, Frank T Bergmann, Andrew Finney, Colin S Gillespie, Tomáš Helikar, Stefan Hoops, Rahuman S Malik‐Sheriff, Stuart L Moodie, Ion I Moraru, Chris J Myers, Aurélien Naldi, Brett G Olivier, Sven Sahle, James C Schaff, Lucian P Smith, Maciej J Swat, Denis Thieffry, Leandro Watanabe, Darren J Wilkinson, Michael L Blinov, Kimberly Begley, James R Faeder, Harold F Gómez, Thomas M Hamm, Yuichiro Inagaki, Wolfram Liebermeister, Allyson L Lister, Daniel Lucio, Eric Mjolsness, Carole J Proctor, Karthik Raman, Nicolas Rodriguez, Clifford A Shaffer, Bruce E Shapiro, Joerg Stelling, Neil Swainston, Naoki Tanimura, John Wagner, Martin Meier‐Schellersheim, Herbert M Sauro, Bernhard Palsson, Hamid Bolouri, Hiroaki Kitano, Akira Funahashi, Henning Hermjakob, John C Doyle, Michael Hucka, SBML Level 3 Community members, Richard R Adams, Nicholas A Allen, Bastian R Angermann, Marco Antoniotti, Gary D Bader, Jan Červený, Mélanie Courtot, Chris D Cox, Piero Dalle Pezze, Emek Demir, William S Denney, Harish Dharuri, Julien Dorier, Dirk Drasdo, Ali Ebrahim, Johannes Eichner, Johan Elf, Lukas Endler, Chris T Evelo, Christoph Flamm, Ronan MT Fleming, Martina Fröhlich, Mihai Glont, Emanuel Gonçalves, Martin Golebiewski, Hovakim Grabski, Alex Gutteridge, Damon Hachmeister, Leonard A Harris, Benjamin D Heavner, Ron Henkel, William S Hlavacek, Bin Hu, Daniel R Hyduke, Hidde de Jong, Nick Juty, Peter D Karp, Jonathan R Karr, Douglas B Kell, Roland Keller, Ilya Kiselev, Steffen Klamt, Edda Klipp, Christian Knüpfer, Fedor Kolpakov, Falko Krause, Martina Kutmon, Camille Laibe, Conor Lawless, Lu Li, Leslie M Loew, Rainer Machne, Yukiko Matsuoka, Pedro Mendes, Huaiyu Mi, Florian Mittag, Pedro T Monteiro, Kedar Nath Natarajan, Poul MF Nielsen, Tramy Nguyen, Alida Palmisano, Jean‐Baptiste Pettit, Thomas Pfau, Robert D Phair, Tomas Radivoyevitch, Johann M Rohwer, Oliver A Ruebenacker, Julio Saez‐Rodriguez, Martin Scharm, Henning Schmidt, Falk Schreiber, Michael Schubert, Roman Schulte, Stuart C Sealfon, Kieran Smallbone, Sylvain Soliman, Melanie I Stefan, Devin P Sullivan, Koichi Takahashi, Bas Teusink, David Tolnay, Ibrahim Vazirabad, Axel von Kamp, Ulrike Wittig, Clemens Wrzodek, Finja Wrzodek, Ioannis Xenarios, Anna Zhukova, Jeremy Zucker

**Affiliations:** ^1^ Computing and Mathematical Sciences California Institute of Technology Pasadena CA USA; ^2^ European Bioinformatics Institute European Molecular Biology Laboratory (EMBL‐EBI) Hinxton UK; ^3^ BioQuant/COS Heidelberg University Heidelberg Germany; ^4^ Medical Informatics Institute for Community Health University Medicine Greifswald Greifswald Germany; ^5^ Institute for Theoretical Biology Humboldt‐University Berlin Berlin Germany; ^6^ Laboratory of Immune System Biology National Institute of Allergy and Infectious Diseases National Institutes of Health Bethesda MD USA; ^7^ Computational Systems Biology of Infection and Antimicrobial‐Resistant Pathogens Institute for Biomedical Informatics (IBMI) University of Tübingen Tübingen Germany; ^8^ Department of Computer Science University of Tübingen Tübingen Germany; ^9^ German Center for Infection Research (DZIF) Tübingen Germany; ^10^ Aix‐Marseille Université CNRS Centrale Marseille Marseille France; ^11^ Instituto Gulbenkian de Ciência Oeiras Portugal; ^12^ ANSYS UK Ltd Milton Park Oxfordshire UK; ^13^ School of Mathematics, Statistics and Physics Newcastle University Newcastle upon Tyne UK; ^14^ Department of Biochemistry University of Nebraska–Lincoln Lincoln NE USA; ^15^ Biocomplexity Institute & Initiative University of Virginia Charlottesville VA USA; ^16^ Eight Pillars Ltd Edinburgh UK; ^17^ Center for Cell Analysis and Modeling UConn Health Farmington CT USA; ^18^ Department of Electrical and Computer Engineering University of Utah Salt Lake City UT USA; ^19^ Institut de Biologie de l'ENS (IBENS) Département de Biologie École Normale Supérieure CNRS INSERM Université PSL Paris France; ^20^ SysBioLab AIMMS Vrije Universiteit Amsterdam Amsterdam the Netherlands; ^21^ Applied BioMath, LLC Concord MA USA; ^22^ Department of Bioengineering University of Washington Seattle WA USA; ^23^ Simcyp (a Certara company) Sheffield South Yorkshire UK; ^24^ The Alan Turing Institute British Library London UK; ^25^ California Institute of Technology Pasadena CA USA; ^26^ Department of Computational and Systems Biology University of Pittsburgh Pittsburgh PA USA; ^27^ Department of Biosystems Science and Engineering ETH Zürich Basel Switzerland; ^28^ Management & IT Consulting Division Mizuho Information & Research Institute, Inc. Tokyo Japan; ^29^ Université Paris‐Saclay INRAE MaIAGE Jouy‐en-Josas France; ^30^ Oxford e‐Research Centre (OeRC) Department of Engineering Science University of Oxford Oxford UK; ^31^ College of Sciences NC State University Raleigh NC USA; ^32^ Department of Computer Science University of California Irvine CA USA; ^33^ Institute of Cellular Medicine Newcastle University Newcastle upon Tyne UK; ^34^ Department of Biotechnology, Bhupat and Jyoti Mehta School of Biosciences Indian Institute of Technology (IIT) Madras Chennai India; ^35^ Initiative for Biological Systems Engineering (IBSE) IIT Madras Chennai India; ^36^ Robert Bosch Centre for Data Science and Artificial Intelligence (RBC‐DSAI) IIT Madras Chennai India; ^37^ The Babraham Institute Cambridge UK; ^38^ Department of Computer Science Virginia Tech Blacksburg VA USA; ^39^ Department of Mathematics California State University Northridge CA USA; ^40^ Department of Biosystems Science and Engineering SIB Swiss Institute of Bioinformatics ETH Zürich Basel Switzerland; ^41^ Institute of Integrative Biology University of Liverpool Liverpool UK; ^42^ Science Solutions Division Mizuho Information & Research Institute, Inc. Tokyo Japan; ^43^ IBM Research Australia Melbourne Vic. Australia; ^44^ Department of Bioengineering University of California San Diego La Jolla CA USA; ^45^ Systems Immunology Benaroya Research Institute at Virginia Mason Seattle WA USA; ^46^ The Systems Biology Institute Tokyo Japan; ^47^ Okinawa Institute of Science and Technology Okinawa Japan; ^48^ Department of Biosciences and Informatics Keio University Yokohama Kanagawa Japan

**Keywords:** computational modeling, file format, interoperability, reproducibility, systems biology, Computational Biology, Metabolism, Methods & Resources

## Abstract

Systems biology has experienced dramatic growth in the number, size, and complexity of computational models. To reproduce simulation results and reuse models, researchers must exchange unambiguous model descriptions. We review the latest edition of the Systems Biology Markup Language (SBML), a format designed for this purpose. A community of modelers and software authors developed SBML Level 3 over the past decade. Its modular form consists of a core suited to representing reaction‐based models and packages that extend the core with features suited to other model types including constraint‐based models, reaction‐diffusion models, logical network models, and rule‐based models. The format leverages two decades of SBML and a rich software ecosystem that transformed how systems biologists build and interact with models. More recently, the rise of multiscale models of whole cells and organs, and new data sources such as single‐cell measurements and live imaging, has precipitated new ways of integrating data with models. We provide our perspectives on the challenges presented by these developments and how SBML Level 3 provides the foundation needed to support this evolution.

## Graphical abstract

Over the past two decades, scientists from different fields have been developing SBML, a standard format for encoding computational models in biology and medicine. This article summarizes recent progress and gives perspectives on emerging challenges.

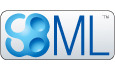

## Introduction

Systems modeling and numerical simulations in biology can be traced to the mid‐20th century. Though general theorizing about systems began earlier, the application of systems analysis to biology gained attention in the 1950s thanks to the work of biologists such as von Bertalanffy and Kacser (Von Bertalanffy, 1950; Kacser, 1957). The era of numerical simulation in biology truly began with the landmark works of Chance on enzyme kinetics (Chance *et al*, 1940), Hodgkin and Huxley on the molecular basis of neuronal transmission (Hodgkin & Huxley, 1952), and Turing on the chemical basis of morphogenesis (Turing, 1952). Since then, the number and variety of models have grown in all of the life sciences. As precise descriptions of phenomena that can be simulated, analyzed, and compared with experimental data, models provide unique insights that can confirm or refute hypotheses, suggest new experiments, and identify refinements to the models.

The availability of more data, more powerful modeling methods, and dramatically increased computing power led to the rise of systems biology as a compelling research theme around the turn of the millennium (Kitano, 2000; Ideker *et al*, 2001). Though computational models were at first published as printed equations in journal articles, the desire to reuse an ever‐increasing number of models called for digital formats that were interoperable between software systems and could be easily exchanged between scientists (topics of interest as early as the 1960s; c.f. Garfinkel, 1969). This drove efforts to create tool‐*independent* ways of representing models that could avoid the potential for human translation errors, be stored in databases, and provide a common starting point for simulations and analyses regardless of the software used (Goddard *et al*, 2001; Hucka *et al*, 2001; Lloyd *et al*, 2004). One such effort was SBML, the Systems Biology Markup Language. Its initial design was motivated by discussions to create a “metabolic model file format” following a 1999 workshop (recounted by Kell & Mendes, 2008). A distributed community thereafter discussed ideas that informed work at Caltech in late 1999/early 2000 and led (after a series of public drafts) to the specification of the official version of SBML Level 1 version 1 being released in March 2001 (Hucka *et al*, 2003).

**Figure 1:**
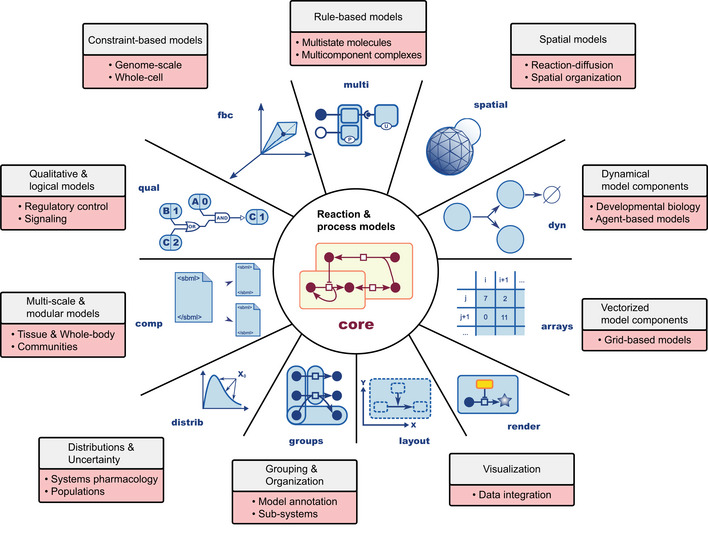
SBML Level 3 (Hucka *et al*, 2019) consists of a core (center) and specialized SBML Level 3 *packages* (in blue), which provide syntactical constructs to support additional modeling approaches The packages support new types of modeling (in the gray boxes) needed for large and complex models such as those used in various domains and fields of biology (in the light red boxes). The meanings of SBML package labels such as “fbc” are given in Table 1, with additional package information in Box  1.

While SBML was initially developed to exchange compartmental models of biochemical reaction networks primarily formulated in terms of chemical kinetics (Hucka *et al*, 2001), it was always understood that there existed more types of models than the initial version of SBML could represent explicitly. However, seeking community consensus on a limited set of simpler features, which could be readily implemented in software at the time, was deemed a more pragmatic strategy. A deliberate decision was taken to delay the addition of more advanced capabilities to a later time. As a result, SBML has evolved in stages in a community‐driven fashion that has benefited from the efforts of many researchers worldwide over two decades. As time passed, the need to support a broader range of model types, modeling frameworks, and research areas became apparent. SBML's success in serving as an interchange format for basic types of models led communities of modelers to ask whether it could be adapted or expanded to support more types. In addition to reaction‐diffusion models, alternative modeling frameworks have risen in popularity in the past decade (Machado *et al*, 2011), and researchers have faced interoperability problems between software tools developed for their use. These needs drove a profound change in SBML's structure: A facility to permit layering the core of SBML with new features suited to more types of models, together with a way for individual models to identify which sets of extensions they need for proper interpretation. The release of SBML Level 3 (Hucka *et al*, 2010) has provided a new foundation to enable the exchange of a greater variety of models in various domains of biology (Fig 1).

In the rest of this article, we begin by summarizing SBML's general structure and then describe the modularity introduced in Level 3 and the wide range of modeling formalisms supported by Level 3 packages. We follow that by describing the community aspects of SBML development. We continue with a discussion of SBML's impact on both computational modeling and the modeling community, and finally, we close with a discussion of forthcoming challenges.

## The structure of SBML

**Figure 2:**
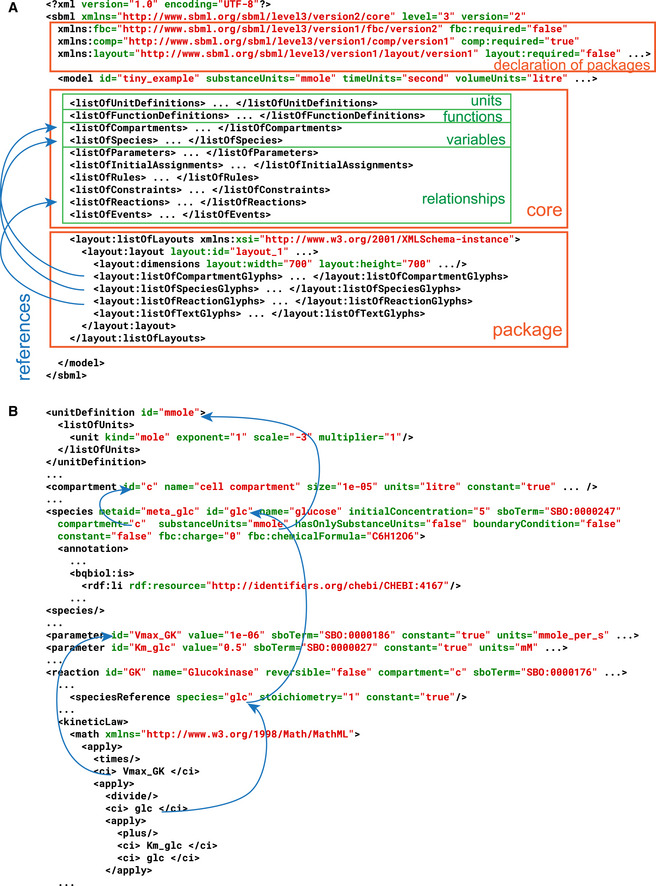
A closer look at SBML (A) Fragments of the global structure of an SBML file. In this example, the use of several SBML packages is declared in the file header. Model elements in the file include the descriptions of model variables, as well as their relationships. Elements of the same type are collected into “ListOf” elements; model parameters are in the ListOfParameters element. SBML package elements can refer to elements in the SBML Core as necessary. (B) Model elements are linked through unique identifiers used in the mathematical constructs and the elements describing the reactions, the molecular species, and their localization. The full model for this example is available in BioModels Database (Malik‐Sheriff *et al*, 2020) as the model with identifier MODEL1904090001.

The core of SBML is focused on encoding models in which entities are located in containers and are acted upon by processes that modify, create, or destroy entities. The containers do not need to correspond to physical structures; they can be conceptual or abstract. Additional constructs allow parameters, initial conditions, other variables, and other mathematical relationships to be defined (Fig 2A). In the most common type of model, the “entities” are biochemical substances, the “containers” are well‐mixed and spatially homogeneous, and the “processes” are biochemical reactions happening within or between the containers. This originally led to the SBML constructs being named *species*,* compartments*, and *reactions*, respectively (Fig 2B), but these names are historical artifacts and belie the generality of the underlying scheme. Software applications can map the names to other concepts to better suit their purposes. For instance, “species” could be mapped to populations of molecules, cells, or even organisms.

Modelers and software developers are encouraged to use SBML's reaction construct to define a model's behavior in preference to formulating the model explicitly as a system of equations. This gives users freedom to convert the model into the final format they prefer—a simpler operation than (for example) inferring a reaction network from a system of differential equations. More importantly, the approach also naturally handles models where reaction kinetics are unknown or unneeded, such as interaction maps, and supports the elaboration of the reaction construct using SBML packages (discussed below). That said, the use of reactions is optional, and SBML provides features sufficient for encoding a large diversity of purely mathematical models, too. Whether using reactions or not, values of model variables and their changes over time may be fixed or determined by mathematical expressions, either before or during simulation, continuously or in response to discrete events, with or without time delays. Units of measurement can be specified for all entities and values; in addition to adding a layer of essential physical knowledge (after all, how else could one interpret whether a time course is in milliseconds or years?), information about units can be used to verify the relationships expressed in a model. Units also facilitate reuse of models and components, interconnection of models, conversion of models between different frameworks, and integration of data with models.

SBML does not dictate which framework must be used to analyze or simulate a model; in fact, it purposefully lacks any explicit way to specify what is done with a model—whether to run simulations or other types of analyses, how to run them, or how to present the results—because externalizing this information enhances model reusability and permits independent innovation in separate but complementary formats. Two of the most popular methods for time‐course simulation are commonly used: one is numerical integration of differential equations created from the reactions and other relationships affecting model variables, and the other is simulating the time evolution of the model as a stochastic system via algorithms such as the one developed by Gillespie (1977). Alternative approaches are also in use, particularly when a model is enhanced with SBML packages.

Any element of an SBML model can be elaborated using machine‐readable metadata as well as human‐readable notes. For metadata, two schemes are supported. The first is direct labeling of SBML elements with terms from the Systems Biology Ontology (SBO; Courtot *et al*, 2011), which allows the mathematical semantics of every element of a model to be precisely specified. The second scheme uses semantic web technologies and provides greater flexibility to capture additional metadata. For instance, a molecular species in a model can be linked to a UniProt entry (The UniProt Consortium, 2017) if it represents a protein, or to ChEBI entry (Hastings *et al*, 2013) if it represents a simple chemical. Gene Ontology terms (GO; Ashburner *et al*, 2000) can be attached to species, compartments, and mathematical elements representing biological processes and functions. Simple provenance data such as identities of creators can be added to facilitate attribution and versioning. To help standardize how annotations are stored, SBML encourages the use of guidelines and resources established for this purpose (Le Novère *et al*, 2005). Finally, software tools can also use annotations to encode tool‐specific data in their own formats, thus providing a way to capture data that might otherwise be lost. Annotations thereby help enrich the meaning of model components, facilitate the understanding and reuse of models, and help software work with SBML more flexibly (Neal *et al*, 2019).

The core features described above have been a backbone of SBML ever since Level 2, even as SBML continued to evolve. The development of the modular Level 3, discussed in the next section, provided an opportunity to rethink and redesign a few other rarely used features. For example, the species *charge* attribute, designed to represent molecular charge, was removed in Level 3 in favor of letting an SBML package introduce more complete support for the relevant concepts.

## SBML Level 3's modularity and breadth

Constant evolution in scientific methods presents challenges for the creation of software tools and standards. One challenge arises because the creation of new standards requires labor, testing, and time. This often causes standardization efforts to lag behind the latest technical developments in a constantly moving field. A second challenge is that users want support for new methods and standards in software tools, which pressures developers to implement support quickly. Combined with the first challenge, it means that sometimes problems with a standard's definition are not discovered until more developers attempt to use it in different situations, which in turn often means that revisions to a standard are needed after it is published. Finally, another challenge is that software development often takes place under resource constraints (funding and time), limiting the scope of work that software developers can undertake—including, sometimes, limiting how many features of a standard they can support in their software.

The SBML community sought to address these challenges by putting in place certain structural features in SBML's development process. The first is the notion of *Levels*. A Level in SBML is an attempt to provide a given set of features for describing models, with higher Levels providing more powerful features. For example, the ability to express discrete events was added to SBML Level 2 but does not exist in Level 1. SBML Levels are mostly upwardly compatible, in the sense that the vast majority of models encoded in Level *n* can be translated to Level *n* + 1. *Versions* are used to introduce refinements to a given Level to account for realizations that come from real‐life use of SBML. Finally, SBML Level 3 introduced an extensible modular architecture consisting of a central set of fixed features (named *SBML Level 3 Core*), and a scheme for adding *packages* that can augment the *Core* by extending existing elements, adding new elements, and adjusting the meaning or scope of elements. A model declares which packages it uses in order to guide its interpretation by software applications. If a software tool detects the presence of packages that it does not support, it may inform users if it cannot work with the model. Together, these three features (Levels, Versions, packages) help address the challenges discussed above: they ease coping with evolution in methods by collecting significant changes into discrete stages (SBML Levels), they help deal with the inevitable need for revisions (Versions within Levels), and they allow developers to limit the feature set they implement (SBML Levels on the one hand, and SBML Level 3 packages on the other).

Packages allow SBML Level 3 (Hucka *et al*, 2019) to represent many model types and characteristics in a more natural way than if they had to be shoehorned into SBML Core constructs exclusively. Twelve packages have been proposed to date (Table 1); eight have been fully developed into consensus specifications and are each used by at least two software implementations (Box 1), and another two have draft specifications in use by software tools. New packages can be developed independently, within dedicated communities, at a pace that suits them. This was the case for logical modeling with the CoLoMoTo community (Naldi *et al*, 2015), constraint‐based modeling within the COBRA community (Heirendt *et al*, 2019), and rule‐based modeling with a community of like‐minded software creators (Faeder *et al*, 2009; Zhang *et al*, 2013; Palmisano *et al*, 2014; Boutillier *et al*, 2018).

**SBML Level 3 packages officially part of the standard**

**Distributions**

The “distrib” package (Smith *et al*, 2020) provides the means to encode information about the distribution and uncertainty of numerical values assigned to a model element. Biological models often contain elements that have inexact numerical values, since they are based on values that are stochastic in nature or data that contains uncertainty; however, core SBML has no direct support for encoding values sampled from distributions. The recently‐finalized “distrib” package adds constructs for sampling of random values from probability distributions and describing uncertainty statistics about element values.

**Hierarchical model composition**

The “comp” package (Smith *et al*, 2015) allows users to build models from other complete models or from model fragments, as a way to manage complexity and construct composite models. “Submodels” can be described within the same SBML file or linked from external files. A submodel can act as a template, and the same definition can be reused multiple times in other models to avoid duplication and enable reuse of parts. The “comp” package also enables submodels to have explicit interfaces (known as *ports*) for optional black‐box encapsulation. Finally, “comp” was designed so that a hierarchical model can be converted into a single SBML model that does not use any “comp” features, making it readable by software that does not directly support the package. The library libSBML (Bornstein *et al*, 2008) provides a facility to do this.

**Flux balance constraints**

The “fbc” package (Olivier & Bergmann, 2018) provides a means of encoding constraint‐based models and optimizations, such as is done in Flux Balance Analysis (Bordbar *et al*, 2014). Constructs in the “fbc” package allow for the definition of a list of objectives for minimization or maximization, as well as flux bounds on reactions and gene‐reaction mappings. Additional information such as chemical formula and charge enable further model analyses, including calculation of reaction mass balances, electron leaks, or implausible sources of matter.

**Groups**

The “groups” package (Hucka & Smith, 2016) provides constructs to describe conceptual relationships between model elements. Groupings can indicate classification, partonomy, or merely a collection of things; a group's meaning can be specified using semantic annotations. Groups have no semantic meaning and cannot influence the mathematical interpretation of an SBML model.

**Multistate, multicomponent, and multicompartment species**

The “multi” package (Zhang and Meier‐Schellersheim, 2018) manages the combinatorics produced by entities either composed of multiple components, such as molecular complexes, or that can exist in multiple states, such as proteins with post‐translational modifications. With the “multi” package, rules can be defined for how reactions depend on the states of the entities and their locations. The package adds syntactic constructs for molecular species types, compartment types, features, binding sites, and bonds. Entire families of molecular complexes sharing certain properties can be defined using patterns created using these constructs.

**Qualitative models**

The “qual” package (Chaouiya *et al*, 2015) provides constructs to encode models whose dynamics can be represented by discrete, reachable states connected by state transitions denoting qualitative updates of model elements. Examples include logical regulatory networks (Boolean or multivalued) and Petri nets. The “qual” package introduces SBML elements to allow the definition of qualitative species, which are used to associate discrete levels of activities with entity pools, as well as transitions, which define the possible changes between states in the transition graph.

**Layout and rendering**

The “layout” (Gauges *et al*, 2015) and “render” (Bergmann *et al*, 2018) packages extend SBML to allow graphical representations of networks or pathways to be stored within SBML files. The “layout” package enables the encoding of positions and sizes of graphical elements such as nodes and lines, while the information about colors, fonts, etc., is defined by the “render” package. This separation presents several advantages. For example, applications can offer multiple styles for visualizing the same layout of a network map. Most of the essential aspects of a network diagram can be expressed using just the “layout” package, and thus tools do not necessarily have to implement a full graphics environment if they do not need to support customizing a diagram's look‐and‐feel.

Several benefits accrue from leveraging SBML as a starting point rather than creating a new, independent format. One is it makes clear where common features overlap. Most computational modeling frameworks in the domain of biology share some common concepts—variables that represent characteristics of different kinds of entities and processes that represent interactions between entities, containers/locations, etc.—and reusing SBML Level 3 Core constructs makes the conceptual similarities explicit. This in turn makes interpretation of models easier (no need to learn new terminology) and reuse simpler (no need to translate between independent formats). Another benefit is that the creators of the format can leverage existing features developed for SBML, such as mechanisms for annotations, rather than spend time developing new approaches to achieving the same goals in a new format. This in turn leads to another benefit: the ability to reuse at least some parts of existing software libraries developed for SBML. It also means that a software application may be able to interpret at least *some* fundamental aspects of a model even if the application is not designed to work with a particular SBML Level 3 package, by virtue of understanding SBML Core (and perhaps other packages used by the model). This improves the potential for model reuse, and benefits model creators and software developers alike. Finally, a common foundation simplifies the creation of multiframework models in which some parts of the model use one formalism and other parts use others [e.g., coupling kinetic models with flux balance analysis; Watanabe *et al*, 2018).

Though this modular approach has benefits, it is not without potential pitfalls. The main risks are fragmentation of the community, and incompatibility of packages due to complex feature dependencies. The SBML community has addressed the former by maintaining communications between package developers; the community processes have such interactions built in. As for the latter, API libraries (see Box 2) can handle *some* combinations of packages and hide some of the complexity. Still, there remain some combinations of packages that are not fully understood, and it remains for future work to define how (if ever) they can be combined for use in a single model.

**Software infrastructure for SBML**

**Table:** 

**Application Programming Interface (API)** Open‐source (LGPL) libraries and code generators help read, write, manipulate, validate, and transform SBML. They support all Levels and Versions of SBML, and all Level 3 packages LibSBML (Bornstein *et al*, 2008) (http://sbml.org/Software/libSBML), written in C++, offers interfaces for C, C++, C#, Java, JavaScript, MATLAB, Octave, Perl, PHP, Python, R, and Ruby JSBML (Rodriguez *et al*, 2015) (http://sbml.org/Software/JSBML) offers a pure Java API Deviser (http://sbml.org/Software/Deviser) generates libSBML code for rapid package prototyping	**Test Suite** The SBML Test Suite (http://sbml.org/Software/SBML_Test_Suite) helps developers implement SBML compatibility and helps users check SBML features supported in software Thousands of test cases for Semantic interpretation of models (for both deterministic and stochastic simulation) Syntactic correctness A graphical front end enables cases to be filtered by Level/Version and type of test An online database allows results to be uploaded and compared with results from other simulators
**Validation Facilities** Validation software can check files for compliance to the definition of SBML, good modeling practices, and consistency of units API libraries include built‐in validation Online validator has simple user interface (http://sbml.org/Facilities/Validator) Web services support software access Validation ensures compliance with: SBML syntax SBML validation rules published as part of each accepted SBML specification	**Conversion Facilities** Converters (http://sbml.org/Software/Converters) can translate some other formats to/from SBML Conversion tools support format conversions from MATLAB, BioPAX, CellML, XPP, SBtab, and others Online services such as SBFC (Rodriguez *et al*, 2016) convert uploaded files to a variety of formats API libraries provide converters between different SBML Levels/Versions and different SBML constructs

**Software Guide**

A catalog (http://sbml.org/SBML_Software_Guide) of software applications, libraries, and online services known to support SBML—over 290 entries to date

1. A tabular interface highlights supported SBML features of each software system.
2. A list interface displays human‐readable summaries of software systems.
3. Software can be added to the list upon request.

**Table 1:**
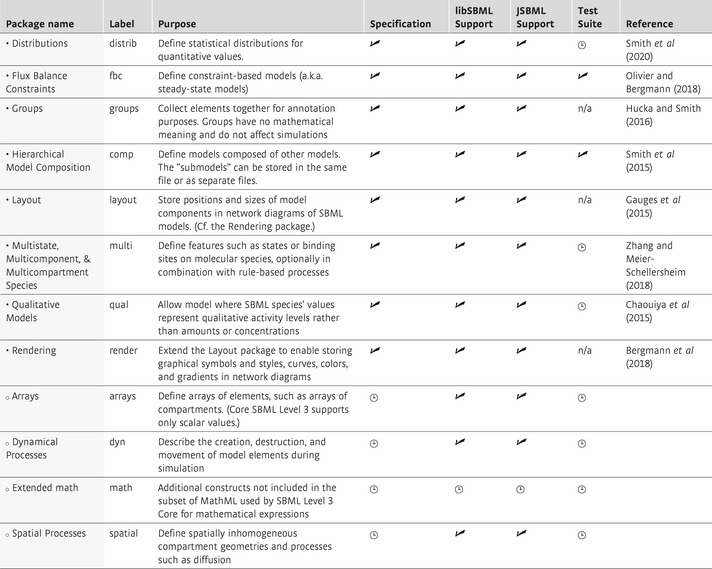
Summary of SBML Level 3 Package statuses. Symbols: ● = released; ○ = not released; ✓ = complete; 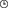  = in progress; n/a = not applicable §

## SBML as a community standard

SBML's success can be attributed largely to its community‐based development and its consensus‐oriented approach. SBML has always been developed through engagement with its user community to achieve goals expressed by that same community. To resolve occasionally conflicting technical demands, a guiding principle has been to seek consensus between different viewpoints and the needs of different groups, to find a middle ground that would be—while perhaps not a perfect solution—an *acceptable* and *usable* solution. This attracted the researchers and software developers who constitute SBML's foremost stakeholders. By using SBML in everything from software to textbooks, they helped drive further development to face the real needs expressed by the people who have those needs. This engagement allowed faster feedback from users to developers and has helped produce a rich toolkit of software and other resources that facilitate SBML's incorporation into software (Box 2).

Over the years, the community has designed rules to organize its governance, develop and maintain the specifications, and facilitate collaboration among users. The development of SBML and its Level 3 packages is shepherded by the SBML Editors, a group of community‐elected volunteers serving terms of 3 years who follow a written and public process detailed on the web portal SBML.org.^1^ SBML Editors write or review SBML specification documents, organize discussions and vote on specific technical issues, and enact the decisions of the community. Major proposed changes to the specifications and packages are discussed by the community via the SBML mailing lists^2^ as well as during annual face‐to‐face meetings.

The community currently comes together twice a year within the context of meetings organized by COMBINE the Computational Modeling in Biology Network; Hucka *et al*, 2015). *HARMONY* (the Hackathon on Resources for Modeling in Biology) is a codefest that focuses on the development of software, in particular via the development of libraries, tools, and specifications; by contrast, the *COMBINE Forum* meetings focus on the presentation of novel tools and the discussion of proposed features. In addition to these general meetings, special SBML working groups are organized as needed to drive SBML package development. COMBINE's central activity is coordinating and harmonizing standardization in computational biology, and SBML is one of its core standards. FAIRsharing, a broader community network that covers life sciences more comprehensively (Sansone *et al*, 2019), maintains interconnected and organized collections of resources in many areas, including curated links between SBML and many associated funders, databases, and standards.^3^

## Impact of SBML

As contributors to developments in methods, software, and standards over the past two decades (Hucka *et al*, 2015), we can attest to SBML's profound impact on the field, both from our own first‐hand experiences and from surveys (Klipp *et al*, 2007) that indicate SBML has become a *de facto* standard. The impact is a result of SBML's community‐oriented development approach and its design.

The SBML development process has helped shape the field partly by directly involving software developers and modelers. Frequent workshops have provided essential feedback for developers to help them better serve modelers’ needs (e.g., Waltemath *et al*, 2014). Workshops as well as resources such as the SBML Software Guide (see Box 2) helped raise awareness of existing tools, which in turn increased their use and the use of SBML. This helped create a culture of sharing models and building on existing work in systems biology (Stanford *et al*, 2015). It also led to new activities centered on the models themselves, including automatic model generation, analysis of model structures, model retrieval, and integration of models with experimental data (Dräger & Palsson, 2014). SBML's successful approach to community organization has led other standardization efforts (BioPAX, NeuroML, SBGN, SED‐ML) to adopt some of the same approaches; SBML was also a founding member of COMBINE (Hucka *et al*, 2015), discussed above. Some of the primary standardization efforts in COMBINE, such as BioPAX (Demir *et al*, 2010) and NeuroML (Gleeson *et al*, 2010), are more domain‐specific than SBML; others, such as CellML (Lloyd *et al*, 2004), overlap SBML's primary domains but offer alternative abstractions; and finally, still others, such as SBGN (van Iersel *et al*, 2012), SBOL (Roehner *et al*, 2016), and SED‐ML (Waltemath *et al*, 2011), are complementary formats.

Before the advent of SBML, it was challenging to exchange models because software tools used incompatible definition schemes. As models increased in size and complexity, manually rewriting them became more difficult, error‐prone, and eventually untenable. The development of SBML has enabled the use of a single model description throughout a project's life cycle even when projects involve heterogeneous software tools (Box 3). SBML‐compatible software tools today allow researchers to use SBML in all aspects of a modeling project, including creation (manual or automated), annotation, comparison, merging, parametrization, simulation/analysis, results comparison, network motif discovery, system identification, omics data integration, visualization, and more. Such use of a standardized format, along with standard annotation schemes (Neal *et al*, 2019) and training in reproducible methods, improves research workflows and is generally recognized as promoting research reproducibility (Waltemath & Wolkenhauer, 2016).

**Examples of SBML use cases**

SBML's impact on computational systems biology includes its facilitation of collaborative work. In multiple instances, it has precipitated entirely new projects, as illustrated by the examples below.

**SBML throughout the model life cycle**

Encoding a model in a standard format such as SBML makes it easier to use different software tools for different purposes without format conversion, and thus makes it easier to leverage the most suitable tools at different points in a workflow. The following is an example. A signaling pathway can be designed graphically using CellDesigner (Funahashi *et al*, 2003). The resulting model can then be semi‐automatically annotated using the online tool semanticSBML (Krause *et al*, 2010). Experimental kinetic information can be retrieved in SBML format from the SABIO‐Reaction Kinetics database (Wittig *et al*,^2017^). Tools such as COPASI (Hoops *et al*, 2006) and PyBioNetFit (Mitra *et al*, 2019) provide facilities to estimate parameters and to simulate the model with various algorithms. Other SBML‐enabled tools such as Tellurium (Medley *et al*, 2018) and PySCeS (Olivier *et al*, 2005) provide capabilities such as identifiability and bifurcation analysis. Each step of the process applied to a model from creation to publication of results—modeling, simulation, and analysis—can be documented using notes attached to every model element. The model can even be turned into a publishable document using SBML2LaTeX (Dräger *et al*, 2009). Finally, the model can be exported from selected modeling tools, together with data and other information all bundled together in COMBINE Archive format (Bergmann *et al*, 2014) and published in model repositories such as BioModels Database (Malik‐Sheriff *et al*, 2020).

**Pipeline for automated model building**

Being able to describe model elements precisely using semantic annotations facilitates the creation of automated pipelines (Dräger *et al*, 2010). Such pipelines can combine existing models with databases of molecular phenotypes or reaction kinetics (Li *et al*, 2010). They can also generate models *de novo* from data resources, as has been demonstrated by the Path2Models project (Büchel *et al*, 2013). Path2Models has produced 143,000 SBML models—all fully annotated—for over 2,600 organisms, by using pathway data. Metabolic pathways were encoded in SBML Level 3 Core while signaling pathways were encoded with the SBML “qual” package (Chaouiya *et al*, 2013). Moreover, constraint‐based models of genome‐scale reconstruction were provided for each organism. Other pipelines have now been built, including ones that can systematically generate alternative models for different tissue types (Wang *et al*, 2012) and patient data (Uhlen *et al*, 2017), an important step toward personalized medicine.

**Development, sharing, and reuse of genome‐scale models of human metabolism**

Constraint‐based modeling approaches such as Flux Balance Analysis and its variants permit the use of whole‐genome reconstructions together with experimental molecular phenotypes, in order to predict how mutations or different environments affect metabolism as well as predict drug targets and biomarkers (O'Brien *et al*, 2015). With the availability of genome‐scale metabolic reconstructions, the use of metabolic flux models at the same scale has been increasing (Bordbar *et al*, 2014). A recent development in the field has been the curation of consensus metabolic models, in particular for human metabolism (Brunk *et al*, 2018). Those community efforts rely on SBML for encoding and sharing the models, including annotations, which are crucial to being able to reuse the reconstructions later, and also for visual representation using the Layout (Gauges *et al*, 2015) and Rendering (Bergmann *et al*, 2018) packages. The Flux Balance Constraint package (Olivier & Bergmann, 2018) enables encoding of the information required for model optimization and flux calculation. Unambiguous encoding in SBML has been shown to be crucial for interpreting models and precisely computing fluxes (Ebrahim *et al*, 2015; Ravikrishnan & Raman, 2015), and new validation tools for genome‐scale metabolic models have been made available by the larger community (e.g., MEMOTE; Lieven *et al*, 2020).

The availability of a well‐defined format has also facilitated the comparison of software tools to each other. Using SBML‐encoded models has become the norm to assess the accuracy of modeling software: initially it is done manually using models from BioModels Database (Bergmann & Sauro, 2008), and now, it is more commonly done using the SBML Test Suite (Box 2). SBML's semantics are defined precisely enough that many simulation systems can produce equivalent results for over 1200 test cases, lending confidence that SBML‐based simulations can be reproducible in different software environments.

While chemical kinetics models have been a staple of systems biology, other modeling frameworks exist. These have benefited from efforts to extend Level 3 to better suit their specific characteristics. Even when models could in principle be encoded using core SBML constructs, the use of features explicitly adapted to the needs of a domain can make model interpretation less error‐prone and more natural. The former issue was demonstrated vividly when *ad hoc* methods of encoding genome‐scale models led to incorrect interpretations, and a subsequent proposal to use SBML Level 3 “fbc” addressed representational inconsistencies that had hindered reproducibility (Ebrahim *et al*, 2015). The use of more domain‐specific forms of encoding has been preferred by several communities, such as the qualitative and rule‐based modeling communities. For example, the quickly adopted package SBML Level 3 “qual” (Chaouiya *et al*, 2015) supports software interoperability for qualitative modeling, illustrated by the use of CellNOpt (Terfve *et al*, 2012), which provides a set of optimal Boolean models that best explains the causal relationships between elements of a signal transduction network and associated data, and the subsequent use of GINsim (Chaouiya *et al*, 2012) or Cell Collective (Helikar *et al*, 2012) to assess the dynamical properties of these models. Rule‐based modeling can represent models that are impossible to express as reaction networks, such as polymerization (Faeder *et al*, 2009), or simply impractical to represent due to the combinatorial number of reactions implied by the rules (Hlavacek *et al*, 2003). Storing rule definitions in SBML is now feasible with the “multi” package, allowing rule‐based modeling tools such as Simmune (Zhang *et al*, 2013) and BioNetGen (Faeder *et al*, 2009) to read and write the same model definitions.

SBML has also eased the automated processing of models to the point where they have become just another type of data in the life sciences. SBML is used today as an import/export format by many databases of mathematical models (Misirli *et al*, 2014; Norsigian *et al*, 2019; Malik‐Sheriff *et al*, 2020), as well as by pathway databases (Caspi *et al*, 2015; Mi *et al*, 2016; Fabregat *et al*, 2017) and reaction databases (Ganter *et al*, 2013; Wittig *et al*, 2017). SBML is the preferred format for model curation in BioModels Database (Malik‐Sheriff *et al*, 2020), not only because of its popularity but also because of its provisions to precisely encode and annotate models to support reproducible modeling. SBML is also used to share models by more generic data management platforms such as SEEK (Wolstencroft *et al*, 2016) and comprehensive online simulation environments (e.g., Moraru *et al*, 2008; Weidemann *et al*, 2008; Lee *et al*, 2009; Peters *et al*, 2017). Moreover, having an agreed‐upon format has facilitated the introduction of better model management strategies. This includes support for tasks such as model storage and retrieval (Henkel *et al*, 2015), version control (Scharm *et al*, 2016b), and checking quality and validity (Liebermeister, 2008; Lieven *et al*, 2020). The proliferation of derived models has led to the development of methods to compare model structure and semantic annotations (Lambusch *et al*, 2018), culminating in the development of several methods to quantify model similarities (Henkel *et al*, 2016), that can then be used to improve the relevance of model searches. Once model elements can be compared, one can align, combine, and merge different models (Krause *et al*, 2010).

A broader impact of SBML as a *de facto* standard has been the support of publishers and funding agencies. Many journals, aware of the challenges surrounding the reproducibility of scientific results, encourage authors not only to describe their models but also to make their models available in electronic form. *Molecular Systems Biology* was the first supporter of submissions in SBML format (beginning in 2005^4^^,^ 5). Today, most journals still avoid *requiring* a specific format, though some such as the BMC^6^ and FEBS^7^ journals do explicitly encourage authors to submit SBML files as supporting material for research where it is relevant. Others, such as Biophysical Journal (Nickerson & Hunter, 2017), recommend authors deposit models in repositories such as BioModels Database, which encourages the use of common standard formats such as SBML. Many funding agencies also now have policies related to data sharing, and some program announcements suggested the use of SBML where appropriate.^8^

Finally, the continued development of SBML has stimulated collaborative work and the creation of consortia. This has led to better awareness and communication within groups interested in specific modeling frameworks. A good example is the CoLoMoTo effort mentioned above; it was launched by researchers who needed a format to exchange qualitative models between their software tools and developed the Qualitative Modeling package for SBML (Naldi *et al*, 2015) as the solution. Nevertheless, challenges remain, as discussed in the next section. These will need to be confronted to ensure the longevity of SBML as well as continued developments.

## Forthcoming challenges

For nearly two decades, SBML has supported mathematical modeling in systems biology by helping to focus the efforts of the community and foster a culture of openness and sharing. The field is evolving rapidly, which presents challenges that the community and SBML must face.

The first challenge is to remain usable in the face of relentless growth in model sizes. One of the drivers of larger size is the rising popularity of genome‐scale metabolic models (Bordbar *et al*, 2014), which can be produced semi‐automatically (Henry *et al*, 2010). Modeling approaches have also been developed to combine the use of several such models (e.g., Bordbar *et al*, 2011). It is reasonable to expect models of ecosystems to be produced soon (microbiomes and their host). Model sizes will also increase as more models of tissues and organs are exchanged and reused, encouraged by the use of software packages that facilitate this approach, such as the open‐source tools CHASTE (Mirams *et al*, 2013) and CompuCell3D (Swat *et al*, 2012). The challenge this presents is how to define, organize, and manage large models. Meeting the challenge will require a combination of novel approaches to model storage (e.g., Henkel *et al*, 2015) and comparison (e.g., Scharm *et al*, 2016a,b), as well as more effective use of SBML Level 3 features. For example, the SBML Hierarchical Model Composition (“comp”) package (Smith *et al*, 2015) provides a way to encode models in SBML out of separate building blocks or from preexisting models; this can make larger models easier to structure and maintain, and it is a natural way to construct multiscale models. Similarly, the Arrays package may help to define and structure larger models by allowing models to be defined in a more compact form.

A related challenge concerns human usability of SBML and similar XML‐based formats. Though SBML is intended for software, not humans, to use directly, desire for a text‐based or spreadsheet‐based equivalent is often voiced (e.g., Kirouac *et al*, 2019). Various answers have been developed in the form of text‐based notations (e.g., Gillespie *et al*, 2006; Smith *et al*, 2009) and spreadsheet conventions (e.g., Lubitz *et al*, 2016), with bidirectional translators for SBML. These formats have undeniable appeal for many users and use cases, despite that they do not capture the entirety of SBML (often having limited or missing facilities to express units, annotations, or SBML packages). Their chief drawback is that they become error‐prone to use as model size increases. Graphical user interfaces (GUIs; e.g., Funahashi *et al*, 2003; Hoops *et al*, 2006; Moraru *et al*, 2008) can overcome this; software with GUIs can help with the cognitive burden of tracking large numbers of model elements. On the other hand, GUIs can be tedious to use when entering large models, performance of some software does not scale well with increasing model sizes, and some cannot be controlled programmatically for automation purposes. A middle ground may be domain‐specific modeling languages layered on top of programming languages such as Python (e.g., Lopez *et al*, 2013; Olivier *et al*, 2005. However, these tend to appeal only to users who are comfortable with (or willing to take time to learn) the programming language used as a substrate. Overall, further innovation in this area would be welcome, both to help support SBML Level 3 packages and to help users cope with ever‐increasing model sizes.

Because of the diversity of biological phenomena amenable to mathematical modeling, as well as their scales and properties, it is likely that a broad variety of modeling approaches will be added to every researcher's essential toolbox (Cvijovic *et al*, 2014). Methods such as multiagent and lattice approaches are coming into wider use to represent evolving cell populations, cell migration, and deformation. Some researchers are experimenting with solutions using existing SBML packages (Watanabe & Myers, 2016; Varela *et al*, 2019). Modeling the development of tissues and organ function may also require combining these approaches with reaction‐diffusion models, or multiphysics approaches (Nickerson *et al*, 2016). Population modeling will need to complement traditional instance‐based systems if we want to take into account patient variability or information coming from single‐cell measurements (Levin *et al*, 1997). The coupling of different approaches within the same simulation experiment is also becoming more frequent. Biomolecular reactions modeled using ODEs, Poisson processes, and Flux Balance Analyses have been coupled in the first whole‐cell model (Karr *et al*, 2015). At the organ level, liver lobules have been modeled using a combination of metabolism and multiagent models (Schliess *et al*, 2014). Several approaches mixing modeling of cell mechanical properties and gene regulatory networks or signaling networks have been used to study morphogenesis (e.g., Tanaka *et al*, 2015). The coupling of different approaches can be done within a single hybrid model, or each model can be simulated using different software and with dynamic synchronization at run time (Mattioni & Le Novère, 2013). Once again, the SBML “comp” package can play a role in supporting these approaches, but other methods and software will be needed in the future, as well as better support for coupling models at run time using, for example, SED‐ML (Waltemath *et al*, 2011).

These developments are arising in a landscape where structural models are sometimes not the central object of study, and instead function as collection of integrated information. An example of this is RECON3D, a comprehensive human metabolic network with metabolite and protein structure information (Brunk *et al*, 2018). SBML will continue to have a pivotal role here too. When SBML was introduced, the state of modeling workflows and software tools was more primitive and it was natural that a model was self‐contained. SBML‐encoded models often had predefined parameter values (as initial values for state variables or parameters for mathematical expressions), but today, modelers increasingly want to use the same model with different parameterizations, sometimes with parameter values expressed as distributions, lists, or ranges rather than unique values. A project may also use an ensemble of related models that differ in parameters or in turning some model elements on or off (Kuepfer *et al*, 2007). The semantic annotation of SBML elements also has become increasingly important, forming a bedrock for many of the analyses using SBML‐encoded models. The growth in size and scope of annotations has recently led the modeling community to propose a standard way of storing annotations in separate linked files (Neal *et al*, 2019), relying on the COMBINE Archive format (Bergmann *et al*, 2014) to bundle everything together. Other formats that can complement SBML have been developed, and further coordination and evolution will undoubtedly happen in the future. As mentioned above, SED‐ML is a format that provides a way to encode what to do with a model, which complements SBML and compensates for its lack of features to define procedures. Finally, experimentation in integrating SBML more directly with other formats and data also continues. For instance, preliminary work has shown that SBML can be enriched with SBOL (Voigt *et al*, 2018) to provide models of DNA components’ behavior (Roehner & Myers, 2014), and conversely, ongoing work in supporting genome‐scale models of metabolism and gene expression (known as *ME‐models*, Thiele *et al*, 2012) augments SBML with SBOL to more fully capture models for use with ME‐modeling software. Future developments in modeling paradigms may require similar flexibility in how models are represented: some may be best served by implementing new SBML packages, others by extending existing packages, still others by combining SBML with other formats.

Besides the technical challenges, social and cultural challenges also exist for formats such as SBML. One is to continue raising awareness among researchers, software developers, and funders of the existence of SBML and related COMBINE standards. Some may not yet be using SBML simply because they are not aware of it, or its recent addition of support for many modeling formalisms (Fig 1). Raising awareness will require continual education and outreach, especially to students and early‐career scientists. Awareness would be aided by greater promotion on the part of journals and reviewers of the use of SBML and related formats in paper submission guidelines. Despite some progress in this area (discussed in the previous section), the lack of stronger demands by journals and reviewers is surely one reason authors are either not aware or not motivated to publish their models in software‐independent formats.

In addition, usability of standard formats depends crucially on their implementation in software tools, and motivating this work is another challenge for SBML. A pivotal factor for the success of SBML has been the extensive software ecosystem, which provides relatively easy import and export of SBML from popular software systems. However, implementing full SBML compatibility in software is not a simple matter, and problems with compatibility in the software ecosystem can be a significant source of frustration. Improving the software requires continuous investment in tool development.

That, in turn, is related to a final challenge: obtaining and maintaining funding. By virtue of not being a native format of any particular software tool, a format such as SBML may require extra work to define by consensus, and then again for developers to implement in software—and still, it will lag behind the leading edge of research because exchange formats only become important after more than one software system has something to exchange. Funders may wonder whether the resources, time and effort spent on standards development would not be better applied to other goals. However, these costs must be weighed against the costs to a whole research field of *not* having standards—and there are many such costs. To take one example, models in nonstandard formats are more difficult to review, verify, and reuse. Journal reviewers may not have access to the necessary software, or the software may not be well tested, all of which increase the chances that the published model contains errors. Researchers can spend substantial time attempting to reproduce the results, only to fail. Worse, this is a repeating cost: failures to reproduce models are rarely published or publicized, which means an untold number of researchers may spend time (and research funding) on a futile effort. Funders recognize that too many research results are irreproducible, and have urged community action (e.g., Collins & Tabak, 2014). The continued development of exchange formats, such as SBML, is a crucial and cost‐effective means to enable reproducible research.

## Conclusion

SBML and associated software libraries and tools have been instrumental in the growth of systems biology. As modeling and simulation grew in popularity, SBML allowed researchers to exchange and (re)use new models in an open, well‐supported, interoperable format. SBML has made possible much of the research pursued by the authors of this article and also helped us to structure our thoughts about our models and the biology they represent. Today, scientists can build, manipulate, annotate, store, reuse, publish, and connect models to each other and to basic data sources. In effect, SBML has turned models into a kind of data and transformed modeling in biology from an art to an exercise in engineering.

As the field of systems biology continues to grow and address emerging challenges, SBML will grow along with it. This evolution will (as it always has) depend on close cooperation between biologists and software developers. We hope that SBML will continue to be a source of inspiration for many researchers, especially those new to the field. In return, may they help develop the next generation of SBML to support more comprehensive, richer, and more diverse models, and expand the reach of systems modeling toward entire cells, organs, and organisms.

## Author contributions

SMK, DW, MK, FZ, AD, CC, and MH wrote the bulk of the manuscript. Together with FTB, AF, CSG, TH, SH, RM‐S, SLM, IIM, CJM, AN, BGO, SS, JCS, LPS, MJS, DT, LW, and DJW, they also wrote and/or edited specifications for SBML Level 3 Core and the Level 3 packages. MLB, KB, JRF, HFG, TMH, YI, WL, ALL, DL, EM, CJP, KR, NR, CAS, BES, JS, JCS, NS, NT, and JW contributed proposals for SBML Level 3 and/or are past or current members of the SBML Team. MM‐S, HMS, BP, HB, HK, AF, HH, JCD, and MH were principal investigators (or the equivalent, depending on the institution) for grants supporting SBML development.

The SBML community members listed in the Appendix supported the development of SBML Level 3 through participation in discussions, commentary on specification documents, and/or implementation of SBML‐using software.

### Conflict of interest

TH has served as a shareholder and/or has consulted for Discovery Collective, Inc.

## Footnotes

1
http://sbml.org/Documents/SBML_Development_Process

2
http://sbml.org/Forums/

3
https://fairsharing.org/FAIRsharing.9qv71f

4
https://www.embo.org/news/press-releases/2005/now-live-molecular-systems-biology-a-first-in-systems-biology-publishing

5
https://www.embopress.org/page/journal/17444292/authorguide#datadeposition

6
https://www.biomedcentral.com/getpublished/writing-resources/additional-files

7
https://onlinelibrary.wiley.com/page/journal/17424658/homepage/ForAuthors.html

8 See, for example, https://grants.nih.gov/grants/guide/pa-files/par-08-023.html

§ Correction added on 4 September 2020, after first online publication: the symbol in row Distributions, column Specification was corrected to a checkmark

## Appendix 1

### Principal authors

Sarah M Keating [S.M.K.] (https://orcid.org/0000‐0002‐3356‐3542) (1) Computing and Mathematical Sciences, California Institute of Technology, Pasadena, California 91125, US; (2) European Bioinformatics Institute, European Molecular Biology Laboratory (EMBL‐EBI), Wellcome Genome Campus, Hinxton, Cambridgeshire, UK; (3) BioQuant/COS, Heidelberg University, Heidelberg 69120, Germany.

Dagmar Waltemath [D.W.] (https://orcid.org/0000-0002-5886-5563) Medical Informatics, Institute for Community Health, University Medicine Greifswald, Greifswald, Germany.

Matthias König [M.K.] (https://orcid.org/0000-0003-1725-179X) Institute for Theoretical Biology, Humboldt‐University Berlin, Berlin, 10115, Germany.

Fengkai Zhang [F.Z.] (https://orcid.org/0000-0001-7112-9328) Laboratory of Immune System Biology, National Institute of Allergy and Infectious Diseases, National Institutes of Health, Bethesda, Maryland 20892, United States.

Andreas Dräger [A.D.] (https://orcid.org/0000-0002-1240-5553) (1) Computational Systems Biology of Infection and Antimicrobial‐Resistant Pathogens, Institute for Biomedical Informatics (IBMI), University of Tübingen, 72076 Tübingen, Germany; (2) Department of Computer Science, University of Tübingen, 72076 Tübingen, Germany; (3) German Center for Infection Research (DZIF), partner site Tübingen, Germany.

Claudine Chaouiya [C.C.] (https://orcid.org/0000-0003-2350-0756) (1) Aix Marseille Univ, CNRS, Centrale Marseille, I2M, Marseille, 13288, France; (2) Instituto Gulbenkian de Ciência, Oeiras, P‐2780‐156, Portugal.

Frank T Bergmann [F.T.B.] (https://orcid.org/0000-0001-5553-4702) BioQUANT/COS, Heidelberg University, Heidelberg, Germany.

Andrew Finney [A.M.F.] ANSYS UK Ltd, United Kingdom.

Colin S Gillespie [C.G.] (https://orcid.org/0000-0003-1787-0275) School of Mathematics, Statistics and Physics, Newcastle University, Newcastle upon Tyne, United Kingdom.

Tomáš Helikar [T.H.] (https://orcid.org/0000-0003-3653-1906) Department of Biochemistry, University of Nebraska – Lincoln, Lincoln, Nebraska 68588, United States.

Stefan Hoops [S.H.] (https://orcid.org/0000-0001-8503-8371) Biocomplexity Institute & Initiative, University of Virginia, 995 Research Park Boulevard, Charlottesville, Virginia 22911, United States.

Rahuman S Malik‐Sheriff [R.M.‐S.] (https://orcid.org/0000-0003-0705-9809) European Bioinformatics Institute, European Molecular Biology Laboratory (EMBL‐EBI), Wellcome Genome Campus, Hinxton, Cambridge CB10 1SD, United Kingdom.

Stuart L Moodie [S.M.] (https://orcid.org/0000-0001-6191-5595) Eight Pillars Ltd, 19 Redford Walk, Edinburgh EH13 0AG, United Kingdom.

Ion I Moraru [I.M.] (https://orcid.org/0000-0002-3746-9676) Center for Cell Analysis and Modeling, UConn Health, Farmington, Connecticut 06030, United States.

Chris J Myers [C.M.] (https://orcid.org/0000-0002-8762-8444) Department of Electrical and Computer Engineering, University of Utah, Salt Lake City, Utah 84112, United States.

Aurélien Naldi [A.N.] (https://orcid.org/0000-0002-6495-2655) Computational Systems Biology Team, Institut de Biologie de l'ENS (IBENS), Département de biologie, École normale supérieure, CNRS, INSERM, Université PSL, 75005 Paris, France.

Brett G Olivier [B.O.] (https://orcid.org/0000-0002-5293-5321) (1) SysBioLab, AIMMS, Vrije Universiteit Amsterdam, De Boelelaan 1085, NL‐1081HV Amsterdam, The Netherlands; (2) BioQuant/COS, Heidelberg University, Heidelberg, 69120, Germany; (3) Computing and Mathematical Sciences, California Institute of Technology, Pasadena, California 91125, United States.

Sven Sahle [S.S.1] COS Heidelberg/BIOQUANT, Heidelberg University, 69120 Heidelberg, Germany.

James C Schaff [J.C.S.] (https://orcid.org/0000-0003-3286-7736) Applied BioMath, LLC, Concord, Massachusetts 01742, United States.

Lucian P Smith [L.S.] (https://orcid.org/0000-0001-7002-6386) (1) Computing and Mathematical Sciences, California Institute of Technology, Pasadena, California, United States; (2) Department of Bioengineering, University of Washington, Seattle, Washington, United States.

Maciej J Swat [M.S.] Simcyp (a Certara company).

Denis Thieffry [D.T.1] (https://orcid.org/0000-0003-0271-1757) Institut de Biologie de l'ENS (IBENS), Département de biologie, École normale supérieure, CNRS, INSERM, Université PSL, 75005 Paris, France.

Leandro Watanabe [L.W.] (https://orcid.org/0000-0001-7030-8690) Department of Electrical and Computer Engineering, University of Utah, Salt Lake City, Utah, 84112, United States.

Darren J Wilkinson [D.J.W.] (https://orcid.org/0000-0003-0736-802X) (1) School of Mathematics, Statistics and Physics, Newcastle University, Newcastle upon Tyne, NE1 7RU, United Kingdom; (2) The Alan Turing Institute, British Library, London, NW1 2DB, United Kingdom.

Michael L Blinov [M.L.B.] (https://orcid.org/0000-0002-9363-9705) Center for Cell Analysis and Modeling, UConn Health, Farmington, Connecticut 06030, United States.

Kimberly Begley [K.B.] (https://orcid.org/0000-0002-1642-7493) Consultant, California Institute of Technology, Pasadena, California 91125, United States.

James R Faeder [J.F.] (https://orcid.org/0000-0001-8127-609X) Department of Computational and Systems Biology, University of Pittsburgh, Pittsburgh, Pennsylvania 15260, United States.

Harold F Gómez [H.G.] Department of Biosystems Science and Engineering, ETH Zürich, Mattenstrasse 26, 4058, Basel, Switzerland.

Thomas M Hamm [T.M.H.] (https://orcid.org/0000-0001-9579-7267) (1) Computational Systems Biology of Infection and Antimicrobial‐Resistant Pathogens, Institute for Biomedical Informatics (IBMI), University of Tübingen, 72076 Tübingen, Germany; (2) Department of Computer Science, University of Tübingen, 72076 Tübingen, Germany.

Yuichiro Inagaki [Y.I.] (https://orcid.org/0000-0003-4011-8487) Management & IT Consulting Division, Mizuho Information & Research Institute, Inc., 2‐3, Kanda‐Nishikicho, Chiyoda‐ku, Tokyo, 101‐8443, Japan.

Wolfram Liebermeister [W.L.] (https://orcid.org/0000-0002-2568-2381) Université Paris‐Saclay, INRAE, MaIAGE, 78350, Jouy‐en‐Josas, France.

Allyson L Lister [A.L.] (https://orcid.org/0000-0002-7702-4495) Oxford e‐Research Centre (OeRC), Department of Engineering Science, University of Oxford, United Kingdom.

Daniel Lucio [D.L.] (https://orcid.org/0000-0002-8912-7213) College of Sciences, North Carolina State University, Raleigh, North Carolina 27695, United States.

Eric Mjolsness [E.M.] (https://orcid.org/0000-0002-9085-9171) Department of Computer Science, University of California, Irvine CA 92697‐3435, United States.

Carole J Proctor [C.P.] (https://orcid.org/0000-0002-1366-1399) Institute of Cellular Medicine, Newcastle University, Newcastle upon Tyne, NE4 5PL, United Kingdom.

Karthik Raman [K.R.] (https://orcid.org/0000-0002-9311-7093) (1) Department of Biotechnology, Bhupat and Jyoti Mehta School of Biosciences, Indian Institute of Technology (IIT) Madras, Chennai ‐ 600 036, India; (2) Initiative for Biological Systems Engineering (IBSE), IIT Madras; (2) Robert Bosch Centre for Data Science and Artificial Intelligence (RBC‐DSAI), IIT Madras.

Nicolas Rodriguez [N.R.] (https://orcid.org/0000-0002-9290-7894) The Babraham Institute, Cambridge, CB22 3AT, United Kingdom.

Clifford A Shaffer [C.S.] (https://orcid.org/0000-0003-0001-0295) Department of Computer Science, Virginia Tech, Blacksburg, Virginia 24061, United States.

Bruce E Shapiro [B.S.] Department of Mathematics, California State University, Northridge, CA 91325, United States.

Joerg Stelling [J.S.] (https://orcid.org/0000-0002-1145-891X) Department of Biosystems Science and Engineering and SIB Swiss Institute of Bioinformatics, ETH Zurich, 4058 Basel, Switzerland.

Neil Swainston [N.S.] (https://orcid.org/0000-0001-7020-1236) Institute of Integrative Biology, University of Liverpool, Liverpool L69 7ZB, United Kingdom.

Naoki Tanimura [N.T.] Science Solutions Division, Mizuho Information & Research Institute, Inc., 2‐3, Kanda‐Nishikicho, Chiyoda‐ku, Tokyo, 101‐8443 Japan.

John Wagner [J.W.] IBM Research Australia, Melbourne, Australia.

Martin Meier‐Schellersheim [M.M.‐S.] (https://orcid.org/0000-0002-8754-6377) Laboratory of Immune System Biology, NIAID, Bethesda, Maryland 20892, United States.

Herbert M Sauro [H.M.S.] (https://orcid.org/0000-0002-3659-6817) University of Washington, Department of Bioengineering, Box 355061, Seattle, WA 98195‐5061.

Bernhard Palsson [B.P.] (https://orcid.org/0000-0003-2357-6785) Department of Bioengineering, University of California, San Diego, La Jolla, California 92093, United States.

Hamid Bolouri [H.B.] Systems Immunology, Benaroya Research Institute at Virginia Mason, 1201 Ninth Avenue, Seattle, Washington 98101, United States.

Hiroaki Kitano [H.K.] (https://orcid.org/0000-0002-3589-1953) (1) The Systems Biology Institute, Tokyo, Japan; (2) Okinawa Institute of Science and Technology, Okinawa, Japan.

Akira Funahashi [A.F.] (https://orcid.org/0000-0003-0605-239X) Department of Biosciences and Informatics, Keio University, Yokohama, Kanagawa 223‐8522, Japan.

Henning Hermjakob [H.H.] (https://orcid.org/0000-0001-8479-0262) European Bioinformatics Institute, European Molecular Biology Laboratory (EMBL‐EBI), Wellcome Genome Campus, Hinxton, Cambridgeshire CB10 1SD, United Kingdom.

John C Doyle [J.C.D.] (https://orcid.org/0000-0002-1828-2486) Computing and Mathematical Sciences, California Institute of Technology, Pasadena, California 91125, United States.

Michael Hucka [M.H.] (https://orcid.org/0000-0001-9105-5960) Computing and Mathematical Sciences, California Institute of Technology, Pasadena, California 91125, United States.

### SBML community members

Richard R Adams [R.A.] (https://orcid.org/0000-0003-1501-0941) ResearchSpace, 24 Fountainhall Road, Edinburgh EH9 2LW, United Kingdom.

Nicholas A Allen [N.A.] Amazon Web Services, Seattle, Washington, 98121, United States.

Bastian R Angermann [B.A.] (https://orcid.org/0000-0002-1328-8790) Computational Biology Unit, Laboratory of Systems Biology, National Institute of Allergy and Infectious Diseases, National Institutes of Health, Bethesda, Maryland 20892, United States.

Marco Antoniotti [M.A.] (https://orcid.org/0000-0002-2823-6838) Dipartimento di Informatica, Sistemistica e Comunicazione, Università degli Studi di Milano‐Bicocca, Milan, Italy.

Gary D Bader [G.B.] (https://orcid.org/0000-0003-0185-8861) The Donnelly Centre, University of Toronto, Toronto M5S 3E1, Canada.

Jan Červený [J.ÄŒ.] (https://orcid.org/0000-0002-5046-3105) (1) Department of Adaptive Biotechnologies, Global Change Research Institute CAS, Brno, 60300, Czech Republic; (2) Systems Biology Laboratory, Faculty of Informatics, Masaryk University, Brno, 60300, Czech Republic; (3) Climate Change Cluster (C3), University of Technology Sydney, Ultimo, NSW 2007, Australia.

Mélanie Courtot [M.C.] (https://orcid.org/0000-0002-9551-6370) European Bioinformatics Institute, European Molecular Biology Laboratory (EMBL‐EBI), Wellcome Genome Campus, Hinxton, Cambridgeshire CB10 1SD, United Kingdom.

Chris D Cox [C.D.C.] (https://orcid.org/0000-0001-9818-5477) Civil and Environmental Engineering, University of Tennessee, Knoxville, Tennessee 37996, United States.

Piero Dalle Pezze [P.D.P.] (https://orcid.org/0000-0003-1695-6763) The Babraham Institute, Babraham Campus, Cambridge, CB22 3AT, United Kingdom.

Emek Demir [E.D.] (https://orcid.org/0000-0002-3663-7113) (1) Department of Molecular and Medical Genetics, Oregon Health and Science University, Portland, Oregon 97239, United States; (2) Computational Biology Program, Oregon Health and Science University, Portland, Oregon 97239, United States.

William S Denney [W.D.] (https://orcid.org/0000-0002-5759-428X) Human Predictions, LLC, Cambridge, Massachusetts 02139, United States.

Harish Dharuri [H.D.] Illumina Inc., Foster City, California 94404, United States.

Julien Dorier [J.D.] (https://orcid.org/0000-0002-6717-2451) Vital‐IT, SIB Swiss Institute of Bioinformatics, 1015 Lausanne, Switzerland.

Dirk Drasdo [D.D.] (https://orcid.org/0000-0002-9314-6281) (1) INRIA de Paris and Sorbonne Universites, UPMC Univ paris 6, LJLL, France; (2) Leibniz Research Centre for Working Environment and Human Factors, Dortmund, Germany; (3) IZBI, University of Leipzig, Germany.

Ali Ebrahim [A.E.] Department of Bioengineering, University of California, San Diego, La Jolla, California 92093, United States.

Johannes Eichner [J.E.] Computational Systems Biology of Infection and Antimicrobial‐Resistant Pathogens, Center for Bioinformatics Tübingen (ZBIT), 72076 Tübingen, Germany.

Johan Elf [J.S.E.] (https://orcid.org/0000-0001-5522-1810) Dept Cell and Molecular Biology, SciLifeLab, Uppsala University, Uppsala, Sweden.

Lukas Endler [L.E.] (https://orcid.org/0000-0002-9115-8756) Institut f. Populationsgenetik, Vetmeduni Wien, 1210 Wien, Austria.

Chris T Evelo [C.E.] (https://orcid.org/0000-0002-5301-3142) (1) Department of Bioinformatics ‐ BiGCaT, NUTRIM, Maastricht University, 6229ER Maastricht, The Netherlands; (2) Maastricht Centre for Systems Biology (MaCSBio), Maastricht University, 6229ER Maastricht, The Netherlands.

Christoph Flamm [C.F.] (https://orcid.org/0000-0001-5500-2415) Department of Theoretical Chemistry, University of Vienna, Vienna 1090, Austria.

Ronan MT Fleming [R.F.] (https://orcid.org/0000-0001-5346-9812) (1) Division of Systems Biomedicine and Pharmacology, Leiden Academic Centre for Drug Research, Faculty of Science, Leiden University, The Netherlands; and (2) School of Medicine, National University of Ireland, Galway, Ireland.

Martina Fröhlich [M.F.] (https://orcid.org/0000-0001-6760-7200) Division of Applied Bioinformatics, DKFZ and NCT Heidelberg, 69120, Heidelberg, Germany.

Mihai Glont [M.G.G.] (https://orcid.org/0000-0002-8211-7581) European Bioinformatics Institute, European Molecular Biology Laboratory (EMBL‐EBI), Wellcome Genome Campus, Hinxton, Cambridge, CB10 1SD, United Kingdom.

Emanuel Gonçalves [E.G.] (https://orcid.org/0000-0002-9967-5205) Wellcome Sanger Institute, Wellcome Genome Campus, Hinxton, United Kingdom.

Martin Golebiewski [M.G.] (https://orcid.org/0000-0002-8683-7084) HITS gGmbH, Schloss‐Wolfsbrunnenweg 35, 69118 Heidelberg, Germany.

Hovakim Grabski [H.G.] (https://orcid.org/0000-0001-6115-9339) Department of Medical Biochemistry and Biotechnology, Institute of Biomedicine and Pharmacy, Russian‐Armenian University, Yerevan 0051, Armenia.

Alex Gutteridge [A.G.] (https://orcid.org/0000-0001-7515-634X) GSK.

Damon Hachmeister [D.G.] CMT, LLC.

Leonard A Harris [L.H.] (https://orcid.org/0000-0003-2112-6940) Biochemistry, Vanderbilt University School of Medicine, Nashville, Tennessee 37232, United States.

Benjamin D Heavner [B.D.H.] (https://orcid.org/0000-0003-2898-9044) Department of Biostatistics, University of Washington, Seattle, Washington 98195, United States.

Ron Henkel [R.H.] (https://orcid.org/0000-0001-6211-2719) Business Information Systems, University of Rostock, 18059 Rostock, Germany.

William S Hlavacek [W.S.H.] (https://orcid.org/0000-0003-4383-8711) Theoretical Division, Los Alamos National Laboratory, Los Alamos, New Mexico 87545, United States.

Bin Hu [B.H.] (https://orcid.org/0000-0002-0278-8466) Clinical Cloud Bioinformatics, LLC, Atlanta, Georgia, United States.

Daniel R Hyduke [D.R.H.] Tegmine Therapeutics, San Francisco, California 94114, United States.

Kaito Ii [K.I.] Department of Bio‐sciences and Informatics, Keio University, Kanagawa, 223‐8522, Japan.

Hidde de Jong [H.d.J.] (https://orcid.org/0000-0002-2226-650X) Univ. Grenoble Alpes, Inria, 38000 Grenoble, France.

Nick Juty [N.J.] (https://orcid.org/0000-0002-2036-8350) School of Computer Science, University of Manchester, Manchester M13 9PL, United Kingdom.

Peter D Karp [P.K.] (https://orcid.org/0000-0002-5876-6418) Bioinformatics Research Group, SRI International, Menlo Park, California 94025, United States.

Jonathan R Karr [J.K.] (https://orcid.org/0000-0002-2605-5080) Icahn Institute and Department of Genetics and Genomic Sciences, Icahn School of Medicine at Mount Sinai, New York, New York, 10029, United States.

Douglas B Kell [D.K.] (https://orcid.org/0000-0001-5838-7963) Department of Biochemistry, Institute of Integrative Biology, Faculty of Health and Life Sciences, University of Liverpool, Crown St, Liverpool L69 7ZB, United Kingdom; (2) The Novo Nordisk Foundation Center for Biosustainability, Building 220, Chemitorvet, Technical University of Denmark, 2800 Kongens Lyngby, Denmark.

Roland Keller [R.K.] University of Tuebingen, Tübingen, Germany.

Ilya Kiselev [I.K.] (https://orcid.org/0000-0003-1057-5986) Institute of Computational Technologies SB RAS, Novosibirsk 630090, Russia.

Steffen Klamt [S.K.] (https://orcid.org/0000-0003-2563-7561) Max Planck Institute for Dynamics of Complex Technical Systems, Magdeburg, Germany.

Edda Klipp [E.K.] (https://orcid.org/0000-0002-0567-7075) Theoretical Biophysics, Institute of Biology, Humboldt‐Universität zu Berlin, 10099 Berlin, Germany.

Christian Knüpfer [C.K.] (https://orcid.org/0000-0002-1323-5445) Institute of Computer Science, Faculty of Mathematics and Computer Science, Friedrich Schiller University Jena, 07743 Jena, Germany.

Fedor Kolpakov [F.A.K.] (https://orcid.org/0000-0002-0396-0256) (1) Laboratory of Bioinformatics, Institute of Computational Technologies of SB RAS, Novosibirsk, 630090, Russian Federation; (2) Biosoft.Ru LLC, Novosibirsk, 630090, Russian Federation.

Falko Krause [F.K.] Theoretische Biophysik, Humboldt‐Universität zu Berlin, 10099 Berlin, Germany.

Martina Kutmon [M.S.‐K.] (https://orcid.org/0000-0002-7699-8191) (1) Department of Bioinformatics ‐ BiGCaT, NUTRIM School of Nutrition and Translational Research in Metabolism, Maastricht University, 6229ER Maastricht, Netherlands; (2) Maastricht Centre for Systems Biology (MaCSBio), Maastricht University, 6229ER Maastricht, Netherlands.

Camille Laibe [C.L.1] (https://orcid.org/0000-0002-4625-743X) European Bioinformatics Institute, European Molecular Biology Laboratory (EMBL‐EBI), Wellcome Genome Campus, Hinxton, Cambridgeshire, CB10 1SD, United Kingdom.

Conor Lawless [C.L.2] (https://orcid.org/0000-0002-4186-8506) Wellcome Centre for Mitochondrial Research, Newcastle University, Framlington Place, Newcastle upon Tyne, NE2 4HH, United Kingdom.

Lu Li [L.L.] (https://orcid.org/0000-0002-6199-522X) Signalling, The Babraham Institute, Cambridge, CB22 3AT, United Kingdom.

Leslie M Loew [L.M.L.] (https://orcid.org/0000-0002-1851-4646) University of Connecticut School of Medicine, Farmington, Connecticut 06030, United States.

Rainer Machne [R.M.] (https://orcid.org/0000-0002-1274-5099) Institute for Synthetic Microbiology, Heinrich Heine University Düsseldorf, Germany.

Yukiko Matsuoka [Y.M.] (https://orcid.org/0000-0002-5171-9096) The Systems Biology Institute, Tokyo, Japan.

Pedro Mendes [P.M.] (https://orcid.org/0000-0001-6507-9168) Center for Quantitative Medicine, University of Connecticut School of Medicine, Farmington, Connecticut 06030, United States.

Huaiyu Mi [H.M.] (https://orcid.org/0000-0001-8721-202X) Division of Bioinformatics, Department of Preventive Medicine, Keck School of Medicine of USC, Los Angenes, CA 90033, United States.

Florian Mittag [F.M.] (https://orcid.org/0000-0002-6472-0221) Tübingen, Germany.

Pedro T Monteiro [P.T.M.] (https://orcid.org/0000-0002-7934-5495) INESC‐ID/Instituto Superior Técnico, Universidade de Lisboa, Rua Alves Redol 9, 1000‐029 Lisbon, Portugal.

Kedar Nath Natarajan [K.N.] (https://orcid.org/0000-0002-9264-1280) University of Southern Denmark, Denmark.

Poul MF Nielsen [P.N.] (https://orcid.org/0000-0002-4704-0179) (1) Auckland Bioengineering Institute, The University of Auckland, Auckland, New Zealand; (2) Department of Engineering Science, The University of Auckland, Auckland, New Zealand.

Tramy Nguyen [T.N.] (https://orcid.org/0000-0002-8035-0259) Electrical and Computer Engineering, University of Utah, Salt Lake City, Utah 84129, United States.

Alida Palmisano [A.P.] (https://orcid.org/0000-0002-1859-3719) (1) Department of Computer Science, Virginia Tech, Blacksburg, Virginia, 24060, United States; (2) Department of Biological Sciences, Virginia Tech, Blacksburg, Virginia, 24060, United States.

Jean‐Baptiste Pettit European Bioinformatics Institute, European Molecular Biology Laboratory (EMBL‐EBI), Wellcome Genome Campus, Hinxton, Cambridgeshire CB10 1SD, United Kingdom.

Thomas Pfau [T.P.] (https://orcid.org/0000-0001-5048-2923) Systems Biology Group, Life Sciences Research Unit, University of Luxembourg, Luxembourg.

Robert D Phair [R.P.] (https://orcid.org/0000-0001-7979-2386) Integrative Bioinformatics, Inc.

Tomas Radivoyevitch [T.R.] (https://orcid.org/0000-0002-9701-1851) Quantitative Health Sciences, Cleveland Clinic, Cleveland, Ohio 44195, United States.

Johann M Rohwer [J.R.] (https://orcid.org/0000-0001-6288-8904) Department of Biochemistry, Stellenbosch University, Stellenbosch 7600, South Africa.

Oliver A Ruebenacker [O.R.] Broad Institute, Massachusetts, United States.

Julio Saez‐Rodriguez [J.S‐R.] (https://orcid.org/0000-0002-8552-8976) (1) Institute for Computational Biomedicine, Heidelberg University, Faculty of Medicine, & Heidelberg University Hospital, Bioquant, Heidelberg, Germany; (2) Joint Research Center for Computational Biomedicine, RWTH Aachen University, Faculty of Medicine, Aachen, Germany.

Martin Scharm [M.S.1] (https://orcid.org/0000-0003-4519-7030) Department of Systems Biology and Bioinformatics, University of Rostock, Rostock, 18051, Germany.

Henning Schmidt [H.S.] IntiQuan GmbH, CH‐4055, Basel, Switzerland.

Falk Schreiber [F.S.] (1) Department of Computer and Information Science, University of Konstanz, Konstanz, 78464, Germany; (2) Faculty of IT, Monash University, Melbourne, Australia.

Michael Schubert [M.S.2] (https://orcid.org/0000-0002-6862-5221) European Molecular Biology Laboratory, European Bioinformatics Institute, Wellcome Genome Campus, Cambridge, CB10 1SD, United Kingdom.

Roman Schulte [R.S] Department of Computer Sience, Eberhard Karls University, Tübingen, 72074 Germany.

Stuart C Sealfon [S.C.S.] (https://orcid.org/0000-0001-5791-1217) Department of Neurology and Friedman Brain Institute, Icahn School of Medicine at Mount Sinai, New York, New York, 10029, United States.

Kieran Smallbone [K.S.] (https://orcid.org/0000-0003-2815-7045) Buxworth, High Peak SK23 7NS, United Kingdom.

Sylvain Soliman [S.S.2] (https://orcid.org/0000-0001-5525-7418) Inria Saclay‐Île de France, Lifeware group, 91120 Palaiseau, France.

Melanie I Stefan [M.I.S.] (https://orcid.org/0000-0002-6086-7357) (1) Edinburgh Medical School: Biomedical Sciences, University of Edinburgh, Edinburgh, United Kingdom; (2) ZJU‐UoE Institute, Zhejiang University School of Medicine, Haining, China.

Devin P Sullivan [D.P.S.] (https://orcid.org/0000-0001-6176-108X) Encodia Inc., San Diego, California, United States.

Koichi Takahashi [K.T.] (https://orcid.org/0000-0002-4235-9914) RIKEN Center for Biosystems Dynamics Research, Suita, Osaka 565‐0874, Japan.

Bas Teusink [B.T.] (https://orcid.org/0000-0003-3929-0423) Amsterdam Institute for Molecules, Medicines and Systems, Vrije Universiteit Amsterdam, 1081HV Amsterdam, The Netherlands.

David Tolnay [D.T.2] (https://orcid.org/0000-0002-5281-6410) Computing and Mathematical Sciences, California Institute of Technology, Pasadena, California 91125, United States.

Ibrahim Vazirabad [I.V.] Diagnostic Laboratories and Blood Research Institute, Blood Center of Wisconsin, Milwaukee, Wisconsin, United States.

Axel von Kamp [A.K.] (https://orcid.org/0000-0002-2956-7815) Analysis and Redesign of Biological Networks, Max Planck Institute for Dynamics of Complex Technical Systems, 39106 Magdeburg, Germany.

Ulrike Wittig [U.W.] (https://orcid.org/0000-0002-9077-5664) Scientific Databases and Visualization, Heidelberg Institute for Theoretical Studies, 69118 Heidelberg, Germany.

Clemens Wrzodek [C.W.] (https://orcid.org/0000-0003-4995-780X) (1) Center for Bioinformatics Tuebingen (ZBIT), University of Tuebingen, Tübingen, Germany; (2) Roche Pharmaceutical Research and Early Development, pRED Informatics, Roche Innovation Center Munich, Nonnenwald 2, 82377 Penzberg, Germany.

Finja Wrzodek [F.W.] (https://orcid.org/0000-0001-5031-1016) (1) Center for Bioinformatics Tuebingen (ZBIT), University of Tuebingen, Tübingen, Germany; (2) Group Informatics, Roche Innovation Center Munich, Nonnenwald 2, 82377 Penzberg, Germany.

Ioannis Xenarios [I.X.] (https://orcid.org/0000-0002-3413-6841) Center for Integrative Genomics, Lausanne, University of Lausanne, Switzerland.

Takahiro G. Yamada [T.Y.] (https://orcid.org/0000-0002-1665-1778) Department of Biosciences and Informatics, Keio University, Yokohama, Kanagawa 223‐8522, Japan.

Anna Zhukova [A.Z.] (https://orcid.org/0000-0003-2200-793) Institut Pasteur – Evolutionary Bioinformatics – C3BI, USR 3756 IP CNRS – Paris, France.

Jeremy Zucker [J.Z.] (https://orcid.org/0000-0002-7276-9009) Computational Biology, Pacific Northwest National Laboratory, Richland, Washington, 99352, United States.
